# *ARID1A* alterations and their clinical significance in cholangiocarcinoma

**DOI:** 10.7717/peerj.10464

**Published:** 2020-12-03

**Authors:** Achira Namjan, Anchalee Techasen, Watcharin Loilome, Prakasit Sa-ngaimwibool, Apinya Jusakul

**Affiliations:** 1 Centre for Research and Development of Medical Diagnostic Laboratories, Faculty of Associated Medical Sciences, Khon Kaen University, Khon Kaen, Thailand; 2 Biomedical Science Program, Graduate School, Khon Kaen University, Khon Kaen, Thailand; 3 Cholangiocarcinoma Research Institute, Faculty of Medicine, Khon Kaen University, Khon Kaen, Thailand; 4 Department of Biochemistry, Faculty of Medicine, Khon Kaen University, Khon Kaen, Thailand; 5 Department of Pathology, Faculty of Medicine, Khon Kaen University, Khon Kaen, Thailand

**Keywords:** Bile duct cancer, BAF250a, Enhancer of zeste homolog 2, SWI/SNF, Sequencing

## Abstract

**Background:**

ARID1A is a member of the SWI/SNF chromatin remodeling complex. It functions as a tumor suppressor and several therapeutic targets in *ARID1A*-mutated cancers are currently under development, including EZH2. A synthetic lethal relationship between ARID1A and EZH2 has been revealed in several tumor entities. Although genomic alterations of *ARID1A* have been described in various cancers, no study has examined correlations between *ARID1A* gene mutation and protein expression with clinicopathologic parameters and prognosis, particularly in liver fluke-related cholangiocarcinoma (Ov-CCA). Here, we investigated the clinical significance of *ARID1A* mutations and protein expression in CCA tissues and determined whether there is a correlation with EZH2 protein expression.

**Methods:**

We evaluated ARID1A and EZH2 immunoreactivity using immunohistochemistry in 98 Ov-CCA with a wide range of clinicopathological features. Somatic mutations of *ARID1A* were analyzed using the ICGC sequencing data in 489 of Ov and non Ov-CCA and assessed prognostic values.

**Results:**

While detecting a loss or reduction of ARID1A expression in 54 cases (55%) in Ov-CCA, ARID1A expression was associated with *ARID1A* mutations (*p* < 0.001, adjusted *p*-value < 0.001). We observed that 12 of 13 tumors (92%) with loss of ARID1A expression had truncating mutations. There were nine of 13 tumors (69%) with loss of ARID1A expression and 25 of 41 tumors (61%) with low ARID1A expression exhibited distant metastasis (*p* = 0.028, adjusted *p*-value = 0.168).* ARID1A* was predominantly mutated in Ov-CCA compared to non Ov-CCA (24% and 14% in Ov-CCA and non Ov-CCA, respectively, *p* = 0.027). There were 36 of 72 (50%) and 52 of 79 (66%) tumors with *ARID1A* mutation showed tumor stage IV and T3/T4, respectively. The significant mutual exclusivity and co-occurrence between *ARID1A* and *TP53/KRAS* mutations were not found in ICGC cohort. In addition, high EZH2 expression, a potential synthetic lethal target in *ARID1A*-mutated tumors, was detected in 49 of 98 Ov-CCA (50%). Importantly, neither ARID1A expression nor *ARID1A* mutations correlated with EZH2 expression in this cohort.

**Conclusion:**

We found that *ARID1A* inactivation, by somatic mutation or by loss of expression, frequently occurs in Ov-CCA. Reduction of ARID1A expression and/or somatic mutation was shown to be associated with CCA progression. These findings suggest that ARID1A may serve as a prognostic biomarker, and thus may be a promising therapeutic target for CCA.

## Introduction

Cholangiocarcinoma (CCA) is the second most common liver cancer that develops along the epithelial bile duct, accounting for 10% to 20% of primary liver cancer (Banales et al., 2016). The incidence and mortality rates of CCA have been rising worldwide in the past decade (Saha et al., 2016). The incidence rate of intrahepatic CCA (ICC) reported in the US increased from 0.44 in 1973 to 1.18 in 2012 cases per 100,000 (Saha et al., 2016). In Europe, ICC has increased by 9% from 1996 to 2008, while mortality from ICC increased by around 9% from 1990 to 2008 (Patel, 2001; Bertuccio et al., 2013). Major risk factors of CCA include liver fluke infection, primary sclerosing cholangitis, hepatolithiasis, and choledochal cysts which result in chronic inflammation along the epithelial of bile ducts (Khan, Toledano & Taylor-Robinson, 2008). Based on the endemic area, *Opisthorchis viverri*-associated CCA (Ov-CCA) has been associated with infestation of Ov. The highest incidence rates of Ov-CCA are in South-East Asia, where endemic areas of liver flukes occur (Sripa & Pairojkul, 2008; Banales et al., 2016), especially in countries lining the Mekong River such as Thailand, Vietnam, and Laos (Sripa et al., 2007). In contrast, the major risk factors of non-liver fluke associated CCA (non Ov-CCA) include primary sclerosing cholangitis and cirrhosis. Currently, surgical resection is the only regular option for treatment. Current 5-year survival rates for CCA after surgery and chemotherapy is around 5% to 15% (Pattanathien et al., 2013; Thunyaharn et al., 2013; Luo et al., 2014; Khuntikeo et al., 2015).

Clinical trials evaluating targeted therapies in unselected CCA populations have shown minimal benefits (Chen et al., 2015). The ASCO guidelines have recommended adjuvant capecitabine as the standard of care for a period of six months following curative resection of biliary tract cancers (Shroff et al., 2019). Recently, cisplatin and gemcitabine have become the recognized reference regimen for first-line treatment in patients with advanced biliary tract cancers (Valle et al., 2017). The median survival of standard chemotherapy using gemcitabine and cisplatin combination remains less than one year (Valle et al., 2010). Thus, finding molecular biomarkers that can be used as targets of therapy and/or predict prognosis in CCA are essential to improve disease management and assist in appropriate therapy.

Growing evidence from molecular genetic studies of CCA has increased our understanding of CCA and has initiated a significant shift towards a more precision medicine-based approach. Previous studies reported relatively high frequencies of potentially actionable mutations in CCA (Ross et al., 2014; Nakamura et al., 2015; Jusakul et al., 2017; Montal et al., 2020). Of note, in the high frequency of somatic mutations in genes associated with chromatin remodeling occurring in CCA (Chan-On et al., 2013; Jiao et al., 2013; Simbolo et al., 2014; Jusakul et al., 2017). Among of these genes, genetic alterations in the *ARID1A* were detected in 7% to 36% of ICC (Chan-On et al., 2013; Jiao et al., 2013; Simbolo et al., 2014; Churi et al., 2014; Jusakul et al., 2017) and 5% to 12% of extrahepatic CCA (ECC) cases (Chan-On et al., 2013; Simbolo et al., 2014; Churi et al., 2014; Nakamura et al., 2015). *ARID1A* (also known as *BAF250A*) encodes a nuclear protein involved in chromatin remodeling. Inactivating mutations in *ARID1A* have been identified in a wide variety of malignancies (Wiegand et al., 2010; Guichard et al., 2012; Wang et al., 2015), suggesting that it functions as a tumor suppressor. Inactivation of *ARID1A* is thought to activate cell cycle progression, thereby contributing to uncontrolled cellular proliferation in cancer cells (Ho & Crabtree, 2010). Interestingly, there is interest in developing therapeutic targets in *ARID 1 A*-mutated cancers, including enhancer of zeste homolog 2 (EZH2) (Alldredge & Eskander, 2017). EZH2 is a histone methyltransferase subunit of a polycomb repressor complex. EZH2 inhibition in *ARID1A* mutated tumors acts in a synthetically lethal manner to suppress cell growth and promote apoptosis, revealing a unique new therapeutic opportunity (Bitler et al., 2015). Clinical trials of EZH2 inhibitors for advanced solid tumors are ongoing and have shown promise in *ARID 1 A*-mutated gastric cancer (Alldredge & Eskander, 2017). Thus, *ARID1A* mutational status or its expression might be a surrogate prognostic predictive biomarker of EZH2 inhibitors.

Although genomic alterations of *ARID1A* have been described in CCA, no study has determined whether there are correlations between *ARID1A* gene mutation and protein expression with clinicopathologic parameters and prognosis, particularly in Ov-CCA. In the present study, we analyzed sequencing data from the International Cancer Genome Consortium (ICGC) of 489 tumors and performed immunohistochemical staining for ARID1A in 98 Ov-CCA which were sequenced in ICGC cohort. We evaluated whether ARID1A expression and mutational status could be a prognostic biomarker for Ov-CCA. To study if ARID1A could be a surrogate biomarker for EZH2 inhibitors in CCA, we evaluated EZH2 protein expression in Ov-CCA. The correlation between alterations of ARID1A and EZH2 expression in matched CCA tissues was evaluated for the first time in this study.

## Materials & Methods

### CCA tissue and mutational data

Ninety-eight paraffin embedded human CCA tissues and clinical data were obtained from Cholangiocarcinoma Research Institute, Khon Kaen University, Khon Kaen, Thailand. All patients signed consent forms. The study was approved by the Ethic Committee for Human Research, Khon Kaen University (HE611195). The primary tumor at the time of resection was staged according to the 7th AJCC.

For mutational analysis, a total of 489 mutational data of bile duct tumors were obtained from the ICGC data portal (Jusakul et al., 2017).

### Immunohistochemistry

The expression and localization of ARID1A and EZH2 in CCA tissues were determined by immunohistochemistry (IHC). The primary antibodies include rabbit polyclonal anti-ARID1A (HPA005456, Sigma-Aldrich, Dorset, UK) and rabbit anti-EZH2 antibody (*36-6300,* Invitrogen, CA, USA). IHC was performed as previously described (Thanan et al., 2020). Briefly, the paraffin-embedded tissues were de-paraffinized in xylene and rehydrated through descending series of ethanol. Sections were treated with 0.01 M sodium citrate, pH 6.0 for 3 mins in pressure cooker for antigen retrieval. After blocking with 0.3% (v/v) hydrogen peroxide in phosphate buffered saline, the sections were incubated with primary antibody: anti-ARID1A (1:250) or anti-EZH2 antibody (1:250) at 4 °C overnight. The sections were incubated with peroxidase-conjugated Envision™ secondary antibody (DAKO, Glostrup, Denmark) at room temperature for 1 h. The reaction products were visualized using 3, 3′-diaminobenzidine tetrahydrochloride substrate kit (Vector, Laboratories, Inc., Burlingame, CA, USA). The sections were counterstained with Mayer’s hematoxylin.

The expression of ARID1A and EZH2 was examined in only bile duct cells in CCA. Localization of cytoplasmic and nuclear staining was scored separately. Tumors were scored positive if tumor cells showed definite nuclear staining and negative if tumor nuclei had no immunoreactivity but other nontumor cells from the same samples showed immunoreactivity. Sections were evaluated using the Immunoreactive score (IR score) and was scored by multiplying of the intensity and frequency of DAB-staining results (Halvorsen et al., 2007). The intensity scored as 0 (negative), 1 (weak), 2 (moderate) to 3 (strong) and proportion of positively stained cells expressed as a percentage categorized as 0 = 0%, 1 +  = 1 − 10%, 2 +  = 11 − 50% and 3 +  = >50%. The intensity and proportion of stained cells were multiplied to produce the final score between 0 and 9. The median of IR score was used to divide CCA patients into two groups as low and high expression. The cut-off values of nuclear ARID1A and EZH2 expression were 3.7 and 3.5, respectively. The cut-off values of cytoplasmic ARID1A and EZH2 expression were 2.8 and 2.7, respectively.

### Statistical analysis

The statistical analysis was carried out using SPSS software (version 19.0). The association between mutational data, protein expression profile and the clinicopathological features of CCA patients were performed using Chi-squared or Fisher’s exact test. Adjusted *p*-values were calculated using Benjamini–Hochberg correction. The survival analysis was determined using Kaplan–Meier estimate with log-rank test. Statistical significance was considered at *p* <  0.05.

## Results

### Decreasing nuclear expression of ARID1A in CCA and its correlation with clinicopathological features

**Figure 1:**
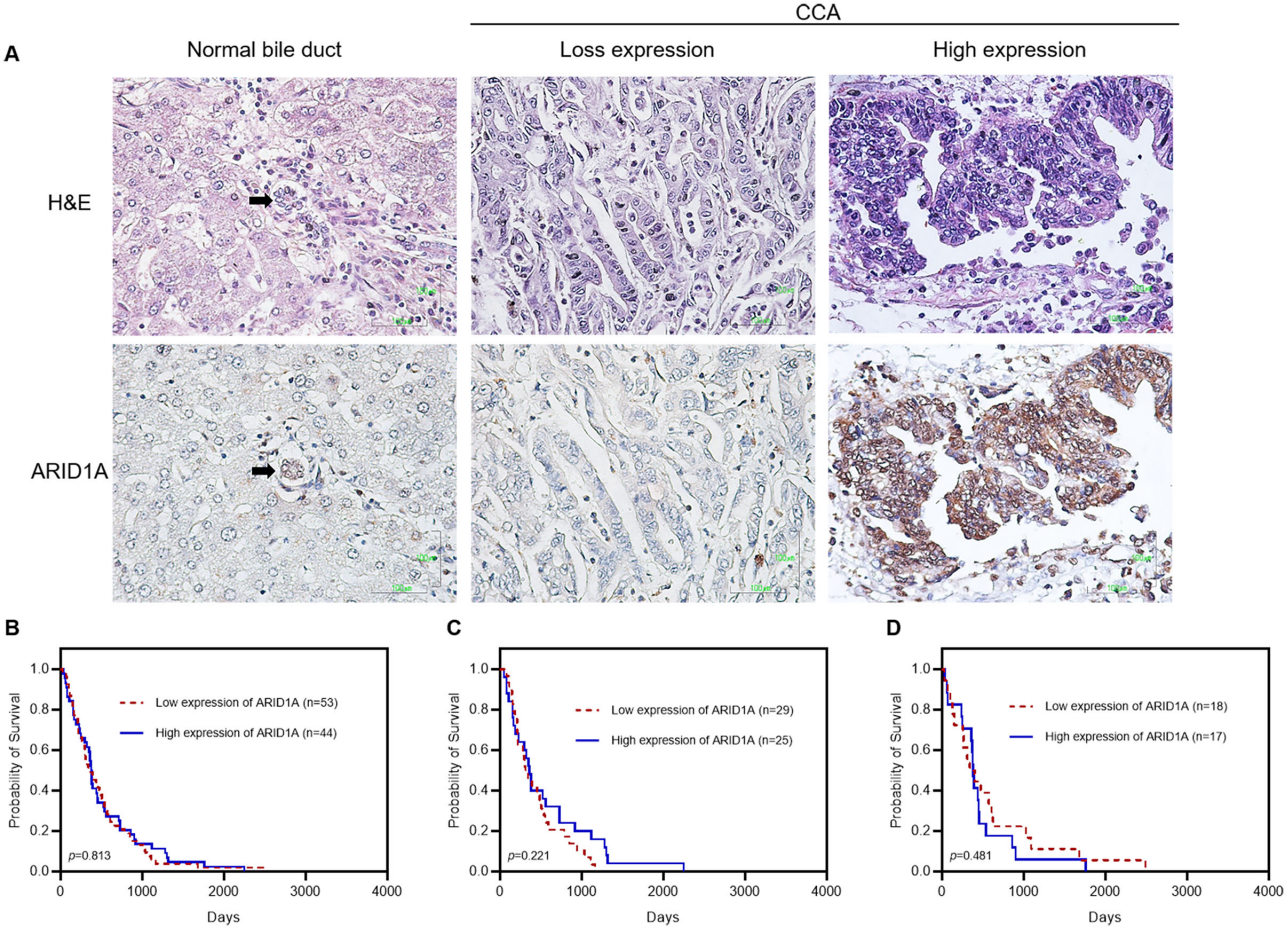
Representative images showing immunohistochemical staining for ARID1A in CCA. (A) Photomicrographs of representative hematoxylin and eosin (H&E) and ARID1A expression in nuclei of normal bile duct (black arrow) and CCA (Original magnification = 400×). (B) Kaplan–Meier analysis for overall survival in CCA. (C) Kaplan–Meier analysis for overall survival in intrahepatic CCA. (D) Kaplan–Meier analysis for overall survival in extrahepatic CCA.

A total of 98 Ov-CCA included in the ICGC cohort (Jusakul et al., 2017), were evaluated for ARID1A expression using IHC. Clinicopathological features are summarized in Table 1. The representative IHC staining of ARID1A in CCA tissue samples is shown in Fig. 1A. Of the total number of cases, 13 (13%) had loss of ARID1A expression, 41 (41%) had low ARID1A expression, and 44 (44%) cases had high ARID1A expression in nuclei. In this study, nuclear expression of ARID1A was not significantly different from matched adjacent normal bile duct. The correlations of nuclear ARID1A protein expression with clinicopathological parameters are shown in Table 2. ARID1A expression tended to associate with distant metastasis. There were 9 of 13 tumors (69%) with loss of ARID1A expression and 25 of 41 tumors (61%) with low ARID1A expression exhibited distant metastasis (*p* = 0.028, adjusted *p*-value = 0.168), suggesting that ARID1A may play a role in CCA progression. Of note, ARID1A expression was associated with *ARID1A* mutations (*p* < 0.001, adjusted *p*-value < 0.001, Table 2 and Fig. S1). We observed that 12 of 13 tumors (92%) with loss of ARID1A expression had truncating mutations (nonsense and frameshift insertion/deletion) (Table 2). In contrast to nuclear expression, there was no correlation between cytoplasmic ARID1A expression and clinicopathological parameters (Table S1). The characteristic of tumors with *ARID1A* truncating mutations is shown in Table S2. Kaplan–Meier survival (Fig. 1B) test showed that ARID1A protein expression in Ov-CCA was not associated with prognosis (HR = 0.953, 95% CI [0.636–1.427]). We stratified CCA based on anatomical subtype: 1) intrahepatic cholangiocarcinoma (ICC) and 2) extrahepatic cholangiocarcinoma (ECC). There was no significant difference in overall survival between groups low and high ARID1A expression in ICC (HR = 0.702, 95% CI [0.397–1.242]) and ECC (HR = 1.278, 95% CI [0.645–2.532]) (Figs. 1C–1D).

**Table 1:** Clinicopathological features of patients.

**Characteristic**		***N*(%)**
Age (years, Mean ±SD)		58 ± 9
Gender	Male/Female	62(63)/36(37)
Staging	0	1(1)
	I	5(5)
	II	13(13)
	III	33(34)
	IV	46(47)
Anatomical subtype	Intrahepatic	54(55)
	Extrahepatic	36(37)
	Extrahepatic/Intrahepatic	8(8)
Distant Metastasis	Positive	50(51)
	Negative	48(49)
Lymph node metastasis	Positive	39(40)
	Negative	59(60)
ARID1A expression	High	44(45)
	Low	54(55)
EZH2 expression	High	49(50)
	Low	49(50)
CCA cases with sequencing data	Ov-related CCA	132(27)
	Non Ov-CCA	357(73)

**Table 2:** Association between nuclear expression of ARID1A and clinicopathological features.

**Clinicopathological features**	**Nuclear ARID1A expression**				
	**Loss n(%)**	**Low n(%)**	**High n(%)**	***p*-value**	**Adjusted** ***p*-value**
**Gender**					
Female	5 (39)	12(29)	19(43)	0.409	0.683
Male	8(61)	29(71)	25(57)		
Total	13	41	44		
**Age, years**					
<58	8 (62)	19(46)	25(57)	0.505	0.683
≥58	5(38)	22(54)	19(43)		
Total	13	41	44		
**Staging**					
0–II	1(8)	10(24)	8(18)	0.437	0.683
III–IV	12(92)	31(76)	36(82)		
Total	13	41	44		
**TNM staging**					
**T factor**					
T1-2	2(15)	16(39)	12(28)	0.233	0.683
T3-4	11(85)	25(61)	31(72)		
**N factor**					
N0	6(46)	19(48)	26(59)	0.502	0.683
N1	7(54)	21(52)	18(41)		
**M factor**					
M0	11(85)	38(93)	38(86)	0.574	0.689
M1	2(15)	3(7)	6(14)		
**Histological type**					
Papillary	6(46)	19(48)	21(48)	1.000	1.000
Non-papillary	7(54)	21(52)	23(52)		
Total	13	40	44		
**Anatomical subtype**					
Intrahepatic	7(58)	22(61)	25(60)	0.982	1.000
Extrahepatic	5(42)	14(39)	17(40)		
Total	12	36	42		
**Distant Metastasis**					
Negative	4(31)	16(39)	28(64)	0.028^*^	0.168
Positive	9(69)	25(61)	16(36)		
Total	13	41	44		
**Lymph node metastasis**					
Negative	8(62)	22(54)	29(66)	0.512	0.683
Positive	5(38)	19(46)	15(34)		
Total	13	41	44		
**ARID1A mutation**					
Wildtype	1(8)	30(73)	38(87)	0.000^*^	
Truncation	12(92)	7(17)	5(11)		0.000^*^
Missense	0(0)	4(10)	1(2)		
Total	13	41	44		
**EZH2 expression**					
Low expression	9(70)	18(44)	22(50)	0.401	0.683
High expression	4(30)	23(56)	22(50)		
Total	13	41	44		

**Notes.**

* *p*-value < 0.05 was considered to indicate statistical significance.

### *ARID1A* mutation and its correlation with clinicopathological features

**Figure 2:**
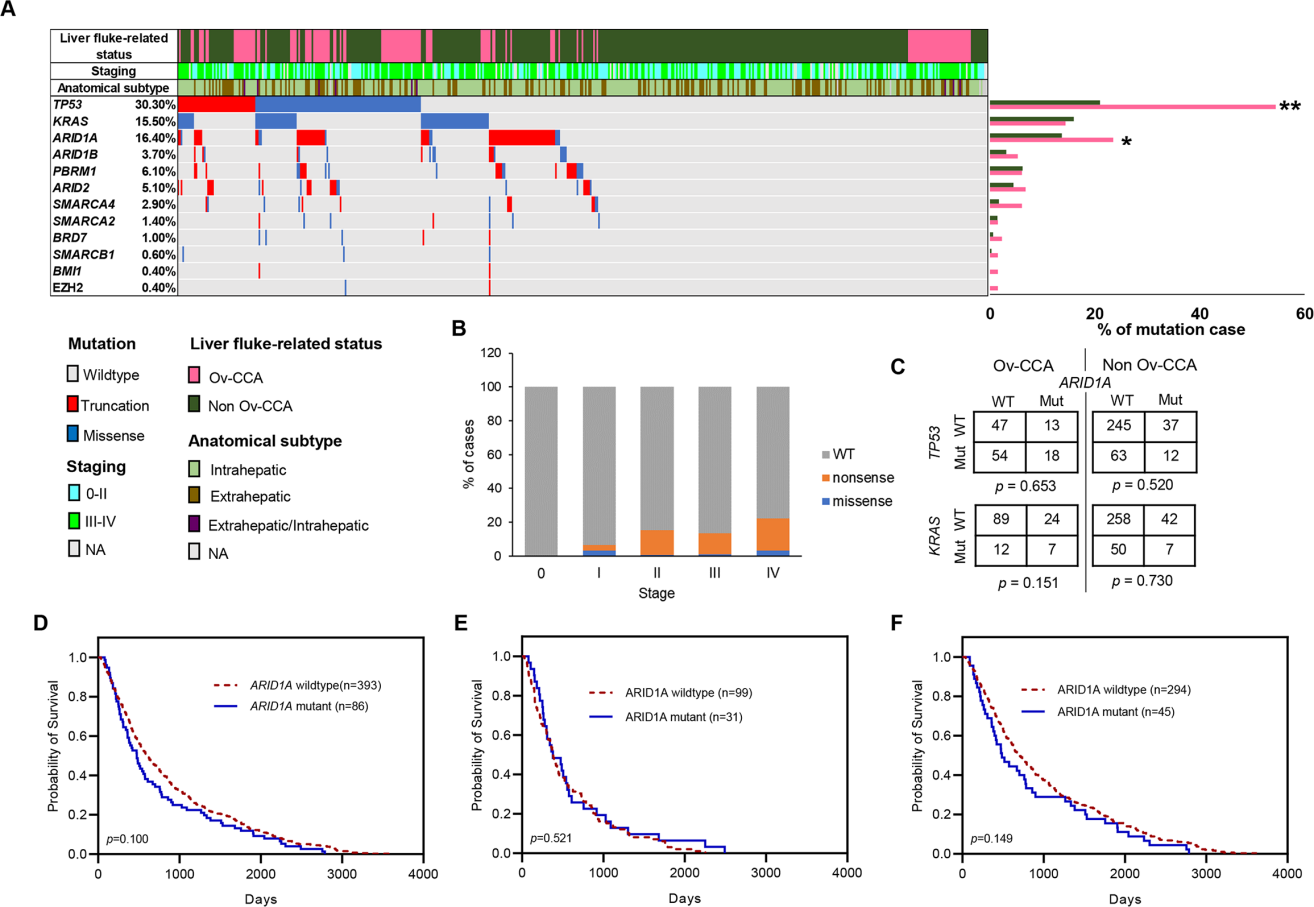
Frequencies of *ARID1A* gene mutation in CCA and its correlation with patient survival. (A) Plot summarizing samples with SWI/SNF subunit, Polycomb complex, *TP53* and *KRAS* gene mutations in the ICGC study. (B) Frequencies of *ARID1A* gene mutations in different stages of CCA. (C) Correlation between *ARID1A* and *TP53/KRAS* mutations. (D) Kaplan–Meier analysis for overall survival in CCA. (E) Kaplan–Meier analysis for overall survival in Ov-CCA. (F) Kaplan–Meier analysis for overall survival in non Ov-CCA harboring *ARID1A* mutations in relation to patients with *ARID1A* wildtype. Mut, Mutant; WT, wildtype. * indicates statistically significant difference (*p* < 0.05, Chi-square test).

Even though the landscape of *ARID1A* mutations has been described in CCA, correlation between *ARID1A* mutations, protein expression and clinical characteristic has not been studied in CCA. To address the clinical impact of *ARID1A* mutations in CCA with different etiologies and clinicopathological features, we performed a systemic analysis of mutational data of *ARID1A* from previous whole genome/exome and targeted sequencing data of 489 CCA (Jusakul et al., 2017) and evaluated the correlation with clinicopathological features. Among a group of genes in SWI/SNF subunit mutated in CCA (Fig. 2A), *ARID1A* was the most frequently mutated gene (80/489; 16%). Most of *ARID1A* mutations were truncating (71/80; 89%), including nonsense (32/71; 45%), frameshift insertion/deletions (39/71; 55%). Interestingly, *ARID1A* was predominantly (*p* = 0.027, Chi-square) mutated in Ov-CCA compared to non Ov-CCA (24% and 14% in Ov-CCA and non Ov-CCA, respectively). As shown in Table 3, *ARID1A* mutations tended to associate with CCA staging (*p* = 0.041, adjusted *p*-value = 0.137), liver fluke related-CCA (*p* = 0.010, adjusted *p*-value = 0.085), and T factor (*p* = 0.017, adjusted *p*-value = 0.085). Of note, there were 36 of 72 (50%) tumors with *ARID1A* gene mutation showed tumor stage IV (Fig. 2B) and 52 of 79 (66%) cases with *ARID1A* mutations presented with T3/T4. Interestingly, 75% (308/409) of *ARID1A* wildtype tumors were non Ov-CCA (*p* = 0.010, adjusted *p*-value = 0.085).

**Table 3:** Association between *ARID1A* mutations and clinicopathological features.

**Clinicopathological features**	***ARID1A*mutation**			
	**Wildtype(%)**	**Mutant(%)**	***p-*value**	**Adjusted*p*-value**
**Gender**				
Female	157(38)	24(30)	0.155	0.388
Male	252(62)	56(70)		
Total	409	80		
**Age, years (mean)**				
<59	111(47)	23(43)	0.52	0.578
≥59	123(53)	31(57)		
Total	234	54		
**Staging**				
0	4(1)	0(0)	0.041^*^	
I	58(15)	4(6)		
II	104(28)	19(26)		0.137
III	84(22)	13(18)		
IV	127(34)	36(50)		
Total	377	72		
**TNM staging**				
**T factor**		
T0-2	187(49)	27(34)	0.017^*^	0.085
T3-4	195(51)	52(66)		
**N factor**				
N0	222(60)	47(65)	0.401	0.501
N1	148(40)	25(35)		
**M factor**				
M0	358(94)	72(95)	0.923	0.923
M1	21(6)	4(5)		
**Liver fluke-related status**				
Positive	101(25)	31(39)	0.010^*^	0.085
Negative	308(75)	49(61)		
Total	409	80		
**Anatomical subtype**				
Intrahepatic	252(64)	55(71)	0.256	0.485
Extrahepatic	143(36)	23(29)		
Total	395	78		
**Distant Metastasis**				
Positive	38(46)	17(55)	0.389	0.501
Negative	45(54)	14(45)		
Total	83	31		
**Lymph node metastasis**				
Positive	33(40)	9(29)	0.291	0.485
Negative	50(60)	22(71)		
Total	83	31		

**Notes.**

* *p*-value <0.05 was considered to indicate statistical significance.

Given the high frequency of *TP53* and *KRAS* mutations in CCA, we performed mutual exclusivity and co-occurrence analysis of *ARID1A* and *TP53/KRAS* mutations. The significant mutual exclusivity and co-occurrence between *ARID1A* and *TP53/KRAS* mutations were not found in the ICGC cohort (Fig. 2C). There was no significant difference in overall survival between *ARID1A* mutated and wildtype CCA (HR = 1.229, 95% CI [0.961–1.573]), Ov-CCA (HR = 0.874, 95% CI [0.579–1.319]) and non Ov-CCA (HR = 1.260, 95% CI [0.919–1.727]) (Figs. 2D–2F).

In non Ov-CCA, we found that 63% (30/48) of *ARID1A* mutant tumors were T3 or T4 and predominantly ICC (40/49, 82%) (Table S3). The association between *ARID1A* mutations and clinicopathological data was not observed in Ov-CCA (Table S4). There was 81% (25/31) of *ARID1A* mutated-Ov-CCA exhibited advanced tumor stage (stage III-IV), but it was not statistically significant (*p* = 0.843).

### EZH2 expression in CCA and its correlation with clinicopathological features

**Figure 3:**
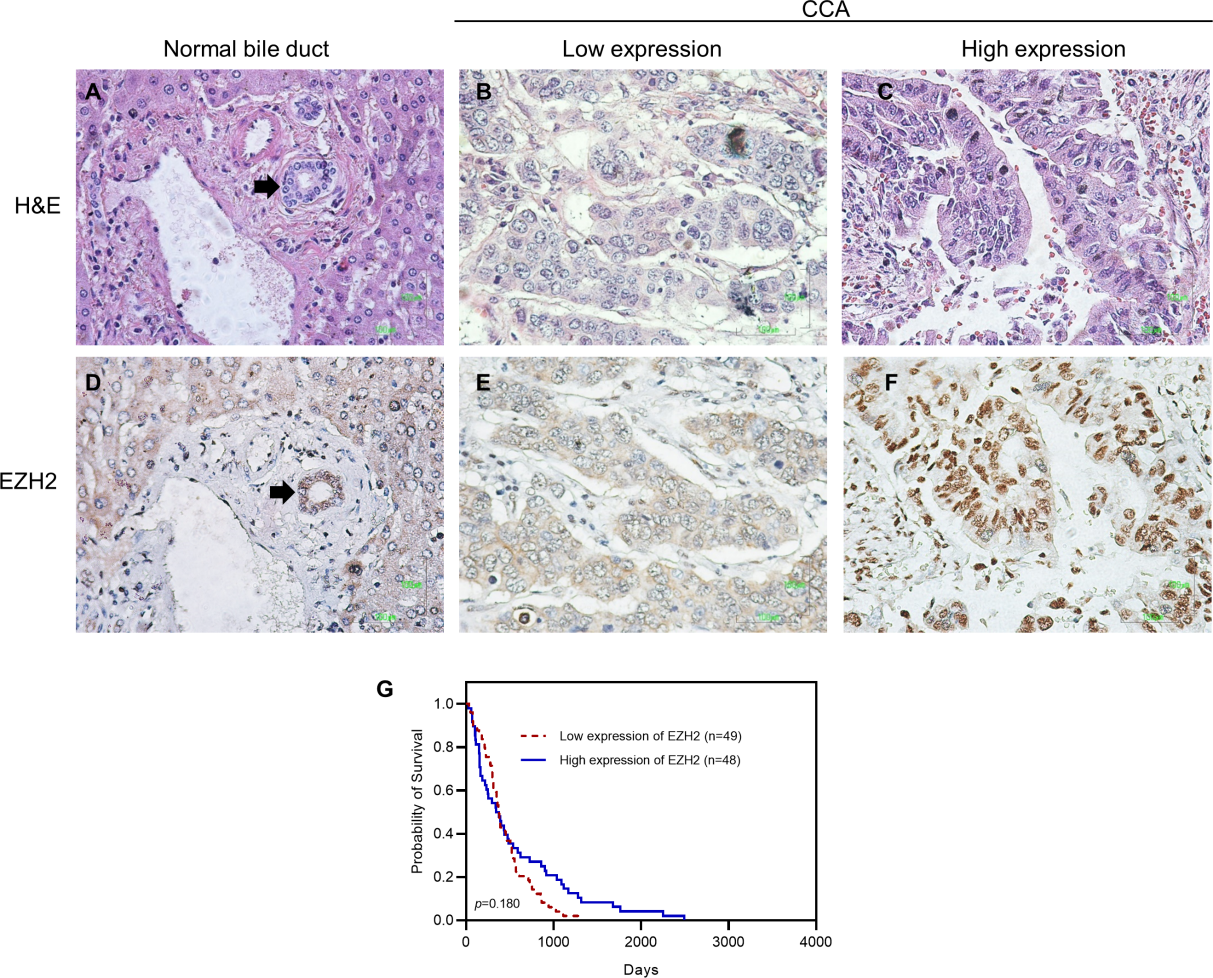
Representative images showing immunohistochemical staining for EZH2 in CCA. (A–C) Photomicrographs of representative hematoxylin and eosin (H&E) in normal bile duct (black arrow) and CCA. (D–F) Photomicrographs of representative EZH2 expression in nuclei of normal bile duct (black arrow) and CCA (Original magnification = 400×). (G) Kaplan–Meier curves indicating survival rate of CCA patients with low and high EZH2 expression. H&E, hematoxylin and eosin.

Several therapeutic targets in *ARID1A* mutated cancers are in development, including EZH2 inhibitors. EZH2 inhibition in *ARID1A* mutated tumors acts in a synthetically lethal manner to inhibit cancer progression, revealing a therapeutic opportunity. Since the response to EZH2 inhibitors correlates with EZH2 overexpression, we then investigated protein expression of EZH2 by IHC staining in tumor cells of Ov-CCA (Fig. 3A). Of the total number of cases, 49 of 98 (50%) had low and 49 (50%) cases had high nuclear expression of EZH2. The significant correlation between nuclear and cytoplasmic EZH2 expression and clinicopathological features was not found in Ov-CCA (Table 4 and Table S1). Additionally, there was no significant difference between groups for low and high expression of EZH2 in overall survival (HR = 0.750, 95% CI [0.491–1.145]) (Fig. 3B). To address if ARID1A alterations could be a predictive biomarker of EZH2 inhibitor, we studied the correlation between EZH2 expression, ARID1A expression and mutation. Neither ARID1A expression nor *ARID1A* mutations associated with EZH2 expression in our cohort (Table 4).

## Discussion

*ARID1A* is one of the most frequently mutated tumor suppressor genes in various types of cancer (Wei et al., 2014). It has been suggested that *ARID1A* mutations and its expression carry prognostic significance (Zhang et al., 2018; Simbolo et al., 2018; Bi et al., 2019). Recently, *ARID1A* has emerged from whole exome and genome studies as one of the significantly mutated gene in CCA (Chan-On et al., 2013; Jusakul et al., 2017)*.* Of note, *ARID1A* mutations were enriched in liver fluke related CCA. However, the prognostic significance of *ARID1A* mutation and its expression has yet been explored in this subgroup of CCA. To date, correlation between *ARID1A* mutations and protein expression in CCA has been explored in the independent studies (Yang et al., 2016; Simbolo et al., 2018; Bi et al., 2019). In this study, we evaluated the clinicopathologic significance of ARID1A protein expression and somatic mutations in the same cohort of Ov-CCA. More importantly, we investigated the correlation between ARID1A expression and *ARID1A* mutations. We found that decreasing ARID1A immunoreactivity in Ov-CCA and loss of ARID1A was associated with tumor metastasis. Our results suggested the somatic mutations of *ARID1A* were associated with immunoreactivity. Additionally, expression of EZH2, a potential synthetic lethal target in *ARID1A* mutated tumors, was also detected in this study.

Regarding the high frequency of *ARID1A* mutations in Ov-CCA, we then evaluated ARID1A expression specifically in Ov-CCA tumors. Here, there were 50% of Ov-CCA exhibited low expression of ARID1A. In this study, there were 9 of 13 CCA (69%) with loss of ARID1A expression and 25 of 41 CCA (61%) with low ARID1A expression exhibited distant metastasis. Similar correlation between ARID1A expression with invasion was reported in ICC (Yang et al., 2016), suggesting that lower ARID1A protein expression is highly correlated with the invasion and metastasis of CCA. Several reports have demonstrated association between loss of ARID1A protein expression and cancer progression in various cancer (Wang et al., 2012; Wei et al., 2014; Zhang et al., 2018). Reduced ARID1A expression was associated with lymph node metastasis, tumor infiltration, and poor prognosis in patients with gastric carcinoma (Wang et al., 2012; Ashizawa et al., 2019). Similarly, ARID1A protein expression was decreased in patient-derived HCC tumor tissues, and that decreased expression was significantly correlated with lymph node and distant metastasis, and poor prognosis (He et al., 2015).

Previous studies have revealed the relevance of *ARID1A* mutation or protein loss to survival in several carcinomas (Ashizawa et al., 2019), although the findings were varied. In this study, *ARID1A* mutation and protein expression were not significantly associated with overall survival of both Ov-CCA and non Ov-CCA. To date, overall survival affected by mutation in *ARID1A* has been shown in ICC (Simbolo et al., 2018). Low expression of ARID1A protein and mRNA were associated with poor prognosis in 57 ICC (Yang et al., 2016). These finding suggest a prognostic role of ARID1A in ICC, unfortunately the correlation between *ARID1A* mutation and protein expression was not determined in that studies. In contrast to ICC, there was no correlation between loss of ARID1A expression and overall survival in ECC (Sasaki et al., 2016). We speculate that these different results may arise from the hypothesis that CCA with different etiologies and anatomical sites display profound differences in their major-driven molecular profiles that drive carcinogenesis (Jusakul et al., 2017). In contrast to several studies, Bi et al. (2019) reported that ARID1A was highly expressed in ICC tumor tissues and increased expression of ARID1A was associated with a higher risk of mortality and disease recurrence in ICC patients. The dual roles in both oncogenicity and tumor suppression of ARID1A were demonstrated in several studies (Otto & Kadoch, 2017; Sun et al., 2017) and may contribute to the difference results between studies. Thus, the functional roles of ARID1A needs to be further investigated.

Loss of ARID1A protein correlated with the presence of *ARID1A* mutations was previously reported in ovarian and uterine endometrioid carcinoma (Wiegand et al., 2010; Guan et al., 2011). All mutations in endometrioid carcinomas were nonsense or insertion/deletion mutations, and there was 73% and 50% of ovarian clear-cell carcinoma and endometrioid carcinoma, respectively, with an *ARID1A* mutation showed a loss of ARID1A expression (Wiegand et al., 2010). In this study, we found the association between ARID1A expression and mutations. Most of CCAs with loss of ARID1A expression had truncating mutation of *ARID1A* gene. Of note, we observed half of CCA with *ARID1A* truncating mutations showed positive protein staining. Our findings are similar to those reported in ovarian clear-cell and endometrioid carcinomas (Wiegand et al., 2010; Guan et al., 2011). It is likely that *ARID1A* mutation occurred in clones of cells within the tumor, resulting in a heterogeneous staining pattern of ARID1A expression. Moreover, the presence of ARID1A immunoreactivity in tumors positive for *ARID1A* mutation may indicate that haploinsufficiency is pathogenic, as has been reported in mice (Gao et al., 2008).

**Table 4:** Association between nuclear EZH2 expression and clinicopathological features.

**Clinicopathological features**	**Nuclear EZH2 expression**			
	**Low n(%)**	**High n(%)**	*p* -value	**Adjusted*p*-value**
**Gender**				
Female	17(35)	19(39)	0.675	0.946
Male	32(65)	30(61)		
Total	49	49		
**Age, years**				
<58	28(57)	24(49)	0.418	0.946
≥58	21(43)	25(51)		
Total	49	49		
**Stages**				
0–II	10(20)	9(18)	0.788	0.946
III–IV	39(80)	40(82)		
Total	49	49		
**TNM staging**				
T factor				
T1-2	15(31)	15(31)	0.946	0.946
T3-4	34(69)	33(69)		
**N factor**				
N0	28(58)	23(47)	0.261	0.946
N1	20(42)	26(53)		
M factor				
M0	43(88)	44(90)	0.749	0.946
M1	6(12)	5(10)		
**Histological type**				
Papillary	23(47)	23(48)	0.923	0.946
Non-papillary	26(53)	25(52)		
Total	49	48		
**Anatomical subtype**				
Intrahepatic	29(67)	25(53)	0.168	0.946
Extrahepatic	14(33)	22(47)		
Total	43	47		
**Distant metastasis**				
Negative	26(53)	22(45)	0.419	0.946
Positive	23(47)	27(55)		
Total	49	49		
**Lymph node metastasis**				
Negative	31(63)	28(57)	0.536	0.946
Positive	18(37)	21(43)		
Total	49	49		
***ARID1A*mutation**				
Wildtype	34(69)	35(71)	0.881	0.946
Truncation	13(27)	11(22)		
Missense	2(4)	3(6)		
Total	49	49		
**ARID1A expression**				
Loss expression	9(18)	4(8)		0.946
Low expression	18(37)	23(47)	0.300	
High expression	22(50)	22(50)		
Total	49	49		

Interestingly, we found that there was 50% and 66% of CCA with *ARID1A* mutation showed tumor stage IV and T3/T4, respectively. Moreover, *ARID1A* was predominantly mutated in Ov-CCA. These data suggest that *ARID1A* mutation may involve in CCA progression and the different etiology may be one of the underlying factors that drives CCA heterogeneity. Sasaki et al. (2016) reported that there was no biliary carcinoma harboring both *ARID1A* and *KRAS* mutations. However, mutually exclusivity between mutations of *ARID1A, TP53* and *KRAS* was not found in this study.

Novel ways of treating patients with *ARID1A* mutations have focused largely on using synthetic-lethal approaches. Bitler et al. (2015) highlighted the potential of targeting the antagonistic activity between SWI/SNF and EZH2 methyltransferase with the EZH2 inhibitor, which triggered apoptosis in *ARID1A*-mutated cells. EZH2 was overexpressed in many solid cancers, suggesting the promise of therapeutic potential of EZH2 inhibitors for cancers (Kim & Roberts, 2016). The response to EZH2 inhibitors often correlates with EZH2 overexpression. There were 50% of Ov-CCA had high expression of EZH2, but the levels of expression did not correlate with patient prognosis. In contrast to our finding, high EZH2 expression was significantly associated with short overall survival in CCA (Wasenang et al., 2019). Neither ARID1A expression nor mutation was correlated with EZH2 expression in our study. Similar to our finding, there was no correlation between ARID1A expression and EZH2 or H3K27me3 amounts in bladder carcinomas. An *in vitro* study showed that ARID1A-depletion did neither increase EZH2 protein or trimethylated H3K27 levels (Garczyk et al., 2018). These finding do not support ARID1A deficiency as predictive biomarker for EZH2-inhibitor treatment response. Future studies should be conducted to validate these preliminary observations by including *ARID1A*-mutated and wildtype CCA cells.

## Conclusions

In conclusion, this is the first investigation that showed the correlation between mutations and expressions of ARID1A within the same Ov-CCA cohort. Based on ARID1A protein expression and mutational analysis, we found that ARID1A inactivation, by somatic mutation or by loss of expression, frequently occurs in Ov-CCA. CCA with distant metastasis had lower ARID1A expression than those without distant metastasis. *ARID1A* mutation may involve in CCA progression and predominantly in CCA tumors with high tumor stage. Importantly, ARID1A protein expression was also correlated with *ARID1A* mutation, suggesting that loss of ARID1A immunoreactivity might be used as a surrogate marker to detect *ARID1A* mutations in tissues. To expand the therapeutic portfolio for CCA patients, EZH2 expression was investigated in Ov-CCA. Neither *ARID1A* mutation nor protein expression correlated with EZH2 expression. Further studies are necessary to determine the role of *ARID1A*-deficiency in response to EZH2 inhibitor in CCA.

##  Supplemental Information

10.7717/peerj.10464/supp-1 Supplemental Information 1 Association between cytoplasmic ARID1A and EZH2 expression and clinicopathological features

10.7717/peerj.10464/supp-2 Supplemental Information 2 Characteristic of tumors with *ARID1A* truncating mutation

10.7717/peerj.10464/supp-3 Supplemental Information 3 Correlation between *ARID1A* mutation with clinicopathological features of Non Ov-related CCA

10.7717/peerj.10464/supp-4 Supplemental Information 4 Correlation between *ARID1A* mutation with clinicopathological features of Ov-CCA

10.7717/peerj.10464/supp-5 Supplemental Information 5 Grouped bar graph of the association between level of ARID1A protein expression and types of ARID1A mutation Wildtype denotes tumors with ARID1A wildtype, Missense denotes tumors with ARID1A missense mutation, Truncation denotes tumors with truncating ARID1A mutation.

10.7717/peerj.10464/supp-6 Supplemental Information 6 Patient Characteristics Raw data of clinicopathological information, *ARID1A* mutations and protein expression level of ARID1A and EAH2.

## Additional Information and Declarations

### Data Availability

The following information was supplied regarding data availability: Representative images for protein staining are available in Figs. 1 and 3. The mutational pattern and clinical information of all cases, correlation analysis between protein expression/mutation and clinicopathological features, characteristics of tumors with ARID1A truncating mutation, and the bar graph of the association between level of ARID1A protein expression and types of ARID1A mutation are available in the Supplemental Files.

## References

[ref-1] Alldredge JK, Eskander RN (2017). EZH2 inhibition in ARID1A mutated clear cell and endometrioid ovarian and endometrioid endometrial cancers. Gynecologic Oncology Research and Practice.

[ref-2] Ashizawa M, Saito M, Min AKT, Ujiie D, Saito K, Sato T, Kikuchi T, Okayama H, Fujita S, Endo H, Sakamoto W, Momma T, Ohki S, Goto A, Kono K (2019). Prognostic role of ARID1A negative expression in gastric cancer. Scientific Reports.

[ref-3] Banales JM, Cardinale V, Carpino G, Marzioni M, Andersen JB, Invernizzi P, Lind GE, Folseraas T, Forbes SJ, Fouassier L, Geier A, Calvisi DF, Mertens JC, Trauner M, Benedetti A, Maroni L, Vaquero J, Macias RIR, Raggi C, Perugorria MJ, Gaudio E, Boberg KM, Marin JJG, Alvaro D (2016). Cholangiocarcinoma: current knowledge and future perspectives consensus statement from the European Network for the Study of Cholangiocarcinoma (ENS-CCA). Nature Reviews Gastroenterology & Hepatology.

[ref-4] Bertuccio P, Bosetti C, Levi F, Decarli A, Negri E, LaVecchia C (2013). A comparison of trends in mortality from primary liver cancer and intrahepatic cholangiocarcinoma in Europe. Annals of Oncology.

[ref-5] Bi C, Liu M, Rong W, Wu F, Zhang Y, Lin S, Liu Y, Wu J, Wang L (2019). High Beclin-1 and ARID1A expression corelates with poor survival and high recurrence in intrahepatic cholangiocarcinoma: a histopathological retrospective study. BMC Cancer.

[ref-6] Bitler BG, Aird KM, Garipov A, Li H, Amatangelo M, Kossenkov AV, Schultz DC, Liu Q, Shih I-M, Conejo-Garcia JR, Speicher DW, Zhang R (2015). Synthetic lethality by targeting EZH2 methyltransferase activity in ARID1A-mutated cancers. Nature Medicine.

[ref-7] Chan-On W, Nairismägi M-L, Ong CK, Lim WK, Dima S, Pairojkul C, Lim KH, McPherson JR, Cutcutache I, Heng HL, Ooi L, Chung A, Chow P, Cheow PC, Lee SY, Choo SP, Tan IBH, Duda D, Nastase A, Myint SS, Wong BH, Gan A, Rajasegaran V, Ng CCY, Nagarajan S, Jusakul A, Zhang S, Vohra P, Yu W, Huang D, Sithithaworn P, Yongvanit P, Wongkham S, Khuntikeo N, Bhudhisawasdi V, Popescu I, Rozen SG, Tan P, Teh BT (2013). Exome sequencing identifies distinct mutational patterns in liver fluke-related and non-infection-related bile duct cancers. Nature Genetics.

[ref-8] Chen JS, Hsu C, Chiang NJ, Tsai CS, Tsou HH, Huang SF, Bai LY, Chang IC, Shiah HS, Ho CL, Yen CJ, Lee KD, Chiu CF, Rau KM, Yu MS, Yang Y, Hsieh RK, Chang JY, Shan YS, Chao Y, Chen LT, Shen W-C, Hsu H-C, Hsu C-H, Shen Y-C, Wang T-E, Li C-P, Chen M-H, Kao W-Y, Chang P-Y, Wu C-C, Teng C-L, Lu C-H, Lin S-J, Wang B-W, Chen Y-Y, Chin Y-H, Chung T-R, Yu W-L, Lee M-H, Lin L-F, Lin P-C, Wu Y-L, Wang H-L, Lu L-J, Chen S-Y, Wu C-C, Wei T-C (2015). A KRAS mutation status-stratified randomized phase II trial of gemcitabine and oxaliplatin alone or in combination with cetuximab in advanced biliary tract cancer. Annals of Oncology.

[ref-9] Churi CR, Shroff R, Wang Y, Rashid A, Kang HC, Weatherly J, Zuo M, Zinner R, Hong D, Meric-Bernstam F, Janku F, Crane CH, Mishra L, Vauthey J-N, Wolff RA, Mills G, Javle M (2014). Mutation profiling in cholangiocarcinoma: prognostic and therapeutic implications. PLOS ONE.

[ref-10] Gao X, Tate P, Hu P, Tjian R, Skarnes WC, Wang Z (2008). ES cell pluripotency and germ-layer formation require the SWI/SNF chromatin remodeling component BAF250a. Proceedings of the National Academy of Sciences of the United States of America.

[ref-11] Garczyk S, Schneider U, Lurje I, Becker K, Vögeli TA, Gaisa NT, Knüchel R (2018). ARID1A-deficiency in urothelial bladder cancer: no predictive biomarker for EZH2-inhibitor treatment response?. PLOS ONE.

[ref-12] Guan B, Mao T-L, Panuganti PK, Kuhn E, Kurman RJ, Maeda D, Chen E, Jeng Y-M, Wang T-L, Shih I-M (2011). Mutation and loss of expression of ARID1A in uterine low-grade endometrioid carcinoma. The American Journal of Surgical Pathology.

[ref-13] Guichard C, Amaddeo G, Imbeaud S, Ladeiro Y, Pelletier L, Maad IB, Calderaro J, Bioulac-Sage P, Letexier M, Degos F, Clément B, Balabaud C, Chevet E, Laurent A, Couchy G, Letouzé E, Calvo F, Zucman-Rossi J (2012). Integrated analysis of somatic mutations and focal copy-number changes identifies key genes and pathways in hepatocellular carcinoma. Nature Genetics.

[ref-14] Halvorsen OJ, Rostad K, Øyan AM, Puntervoll H, Bø TH, Stordrange L, Olsen S, Haukaas SA, Hood L, Jonassen I, Kalland K-H, Akslen LA (2007). Increased expression of SIM2-s protein is a novel marker of aggressive prostate cancer. Clinical Cancer Research.

[ref-15] He F, Li J, Xu J, Zhang S, Xu Y, Zhao W, Yin Z, Wang X (2015). Decreased expression of ARID1A associates with poor prognosis and promotes metastases of hepatocellular carcinoma. Journal of Experimental & Clinical Cancer Research.

[ref-16] Ho L, Crabtree GR (2010). Chromatin remodelling during development. Nature.

[ref-17] Jiao Y, Pawlik TM, Anders RA, Selaru FM, Streppel MM, Lucas DJ, Niknafs N, Guthrie VB, Maitra A, Argani P, Offerhaus GJA, Roa JC, Roberts LR, Gores GJ, Popescu I, Alexandrescu ST, Dima S, Fassan M, Simbolo M, Mafficini A, Capelli P, Lawlor RT, Ruzzenente A, Guglielmi A, Tortora G, Braud Fde, Scarpa A, Jarnagin W, Klimstra D, Karchin R, Velculescu VE, Hruban RH, Vogelstein B, Kinzler KW, Papadopoulos N, Wood LD (2013). Exome sequencing identifies frequent inactivating mutations in BAP1, ARID1A and PBRM1 in intrahepatic cholangiocarcinomas. Nature Genetics.

[ref-18] Jusakul A, Cutcutache I, Yong CH, Lim JQ, Huang MN, Padmanabhan N, Nellore V, Kongpetch S, Ng AWT, Ng LM, Choo SP, Myint SS, Thanan R, Nagarajan S, Lim WK, Ng CCY, Boot A, Liu M, Ong CK, Rajasegaran V, Lie S, Lim AST, Lim TH, Tan J, Loh JL, McPherson JR, Khuntikeo N, Bhudhisawasdi V, Yongvanit P, Wongkham S, Totoki Y, Nakamura H, Arai Y, Yamasaki S, Chow PK-H, Chung AYF, Ooi LLPJ, Lim KH, Dima S, Duda DG, Popescu I, Broet P, Hsieh S-Y, Yu M-C, Scarpa A, Lai J, Luo D-X, Carvalho AL, Vettore AL, Rhee H, Park YN, Alexandrov LB, Gordân R, Rozen SG, Shibata T, Pairojkul C, Teh BT, Tan P (2017). Whole-genome and epigenomic landscapes of etiologically distinct subtypes of cholangiocarcinoma. Cancer Discovery.

[ref-19] Khan SA, Toledano MB, Taylor-Robinson SD (2008). Epidemiology risk factors, and pathogenesis of cholangiocarcinoma. HPB.

[ref-20] Khuntikeo N, Chamadol N, Yongvanit P, Loilome W, Namwat N, Sithithaworn P, Andrews RH, Petney TN, Promthet S, Thinkhamrop K, Tawarungruang C, Thinkhamrop B (2015). Cohort profile: cholangiocarcinoma screening and care program (CASCAP). BMC Cancer.

[ref-21] Kim KH, Roberts CWM (2016). Targeting EZH2 in cancer. Nature Medicine.

[ref-22] Luo X, Yuan L, Wang Y, Ge R, Sun Y, Wei G (2014). Survival outcomes and prognostic factors of surgical therapy for all potentially resectable intrahepatic cholangiocarcinoma: a large single-center Cohort study. Journal of Gastrointestinal Surgery.

[ref-23] Montal R, Sia D, Montironi C, Leow WQ, Esteban-Fabró R, Pinyol R, Torres-Martin M, Bassaganyas L, Moeini A, Peix J, Cabellos L, Maeda M, Villacorta-Martin C, Tabrizian P, Rodriguez-Carunchio L, Castellano G, Sempoux C, Minguez B, Pawlik TM, Labgaa I, Roberts LR, Sole M, Fiel MI, Thung S, Fuster J, Roayaie S, Villanueva A, Schwartz M, Llovet JM (2020). Molecular classification and therapeutic targets in extrahepatic cholangiocarcinoma. Journal of Hepatology.

[ref-24] Nakamura H, Arai Y, Totoki Y, Shirota T, Elzawahry A, Kato M, Hama N, Hosoda F, Urushidate T, Ohashi S, Hiraoka N, Ojima H, Shimada K, Okusaka T, Kosuge T, Miyagawa S, Shibata T (2015). Genomic spectra of biliary tract cancer. Nature Genetics.

[ref-25] Otto JE, Kadoch C (2017). A two-faced mSWI/SNF subunit: dual roles for ARID1A in tumor suppression and oncogenicity in the liver. Cancer Cell.

[ref-26] Patel T (2001). Increasing incidence and mortality of primary intrahepatic cholangiocarcinoma in the United States. Hepatology.

[ref-27] Pattanathien P, Khuntikeo N, Promthet S, Kamsa-ard S (2013). Survival rate of extrahepatic cholangiocarcinoma patients after surgical treatment in Thailand. Asian Pacific Journal of Cancer Prevention.

[ref-28] Ross JS, Wang K, Gay L, Al-Rohil R, Rand JV, Jones DM, Lee HJ, Sheehan CE, Otto GA, Palmer G, Yelensky R, Lipson D, Morosini D, Hawryluk M, Catenacci DVT, Miller VA, Churi C, Ali S, Stephens PJ (2014). New routes to targeted therapy of intrahepatic cholangiocarcinomas revealed by next-generation sequencing. The Oncologist.

[ref-29] Saha SK, Zhu AX, Fuchs CS, Brooks GA (2016). Forty-year trends in cholangiocarcinoma incidence in the U.S.: intrahepatic disease on the rise. The Oncologist.

[ref-30] Sasaki M, Nitta T, Sato Y, Nakanuma Y (2016). Loss of ARID1A expression presents a novel pathway of carcinogenesis in biliary carcinomas. American Journal of Clinical Pathology.

[ref-31] Shroff RT, Kennedy EB, Bachini M, Bekaii-Saab T, Crane C, Edeline J, El-Khoueiry A, Feng M, Katz MHG, Primrose J, Soares HP, Valle J, Maithel SK (2019). Adjuvant therapy for resected biliary tract cancer: ASCO clinical practice guideline. Journal of Clinical Oncology.

[ref-32] Simbolo M, Fassan M, Ruzzenente A, Mafficini A, Wood LD, Corbo V, Melisi D, Malleo G, Vicentini C, Malpeli G, Antonello D, Sperandio N, Capelli P, Tomezzoli A, Iacono C, Lawlor RT, Bassi C, Hruban RH, Guglielmi A, Tortora G, de BraudF, Scarpa A (2014). Multigene mutational profiling of cholangiocarcinomas identifies actionable molecular subgroups. Oncotarget.

[ref-33] Simbolo M, Vicentini C, Ruzzenente A, Brunelli M, Conci S, Fassan M, Mafficini A, Rusev B, Corbo V, Capelli P, Bria E, Pedron S, Turri G, Lawlor RT, Tortora G, Bassi C, Guglielmi A, Scarpa A (2018). Genetic alterations analysis in prognostic stratified groups identified TP53 and ARID1A as poor clinical performance markers in intrahepatic cholangiocarcinoma. Scientific Reports.

[ref-34] Sripa B, Kaewkes S, Sithithaworn P, Mairiang E, Laha T, Smout M, Pairojkul C, Bhudhisawasdi V, Tesana S, Thinkamrop B, Bethony JM, Loukas A, Brindley PJ (2007). Liver fluke induces cholangiocarcinoma. PLOS Medicine.

[ref-35] Sripa B, Pairojkul C (2008). Cholangiocarcinoma: lessons from Thailand. Current Opinion in Gastroenterology.

[ref-36] Sun X, Wang SC, Wei Y, Luo X, Jia Y, Li L, Gopal P, Zhu M, Nassour I, Chuang J-C, Maples T, Celen C, Nguyen LH, Wu L, Fu S, Li W, Hui L, Tian F, Ji Y, Zhang S, Sorouri M, Hwang TH, Letzig L, James L, Wang Z, Yopp AC, Singal AG, Zhu H (2017). Arid1a has context-dependent oncogenic and tumor suppressor functions in liver cancer. Cancer Cell.

[ref-37] Thanan R, Kaewlert W, Sakonsinsiri C, Chaiprasert T, Armartmuntree N, Muengsaen D, Techasen A, Klanrit P, Lert-itthiporn W, Pinlaor S, Pairojkul C (2020). Opposing roles of FoxA1 and FoxA3 in intrahepatic cholangiocarcinoma progression. International Journal of Molecular Sciences.

[ref-38] Thunyaharn N, Promthet S, Wiangnon S, Suwanrungruang K, Kamsa-ard S (2013). Survival of cholangiocarcinoma patients in Northeastern Thailand after supportive treatment. Asian Pacific Journal of Cancer Prevention.

[ref-39] Valle JW, Lamarca A, Goyal L, Barriuso J, Zhu AX (2017). New horizons for precision medicine in biliary tract cancers. Cancer Discovery.

[ref-40] Valle J, Wasan H, Palmer DH, Cunningham D, Anthoney A, Maraveyas A, Madhusudan S, Iveson T, Hughes S, Pereira SP, Roughton M, Bridgewater J, Investigators ABC-02Trial (2010). Cisplatin plus gemcitabine versus gemcitabine for biliary tract cancer. The New England Journal of Medicine.

[ref-41] Wang D, Chen Y, Pan K, Wang W, Chen S, Chen J, Zhao J, Lv L, Pan Q, Li Y, Wang Q, Huang L, Ke M, He J, Xia J (2012). Decreased expression of the ARID1A gene is associated with poor prognosis in primary gastric cancer. PLOS ONE.

[ref-42] Wang N, Xia S, Chen K, Xiang X, Zhu A (2015). Genetic alteration regulated by microRNAs in biliary tract cancers. Critical Reviews in Oncology/Hematology.

[ref-43] Wasenang W, Puapairoj A, Settasatian C, Proungvitaya S, Limpaiboon T (2019). Overexpression of polycomb repressive complex 2 key components EZH2/SUZ12/EED as an unfavorable prognostic marker in cholangiocarcinoma. Pathology - Research and Practice.

[ref-44] Wei X-L, Wang D-S, Xi S-Y, Wu W-J, Chen D-L, Zeng Z-L, Wang R-Y, Huang Y-X, Jin Y, Wang F, Qiu M-Z, Luo H-Y, Zhang D-S, Xu R-H (2014). Clinicopathologic and prognostic relevance of ARID1A protein loss in colorectal cancer. World Journal of Gastroenterology.

[ref-45] Wiegand KC, Shah SP, Al-Agha OM, Zhao Y, Tse K, Zeng T, Senz J, McConechy MK, Anglesio MS, Kalloger SE, Yang W, Heravi-Moussavi A, Giuliany R, Chow C, Fee J, Zayed A, Prentice L, Melnyk N, Turashvili G, Delaney AD, Madore J, Yip S, McPherson AW, Ha G, Bell L, Fereday S, Tam A, Galletta L, Tonin PN, Provencher D, Miller D, Jones SJM, Moore RA, Morin GB, Oloumi A, Boyd N, Aparicio SA, Shih I-M, Mes-Masson A-M, Bowtell DD, Hirst M, Gilks B, Marra MA, Huntsman DG (2010). ARID1A mutations in endometriosis-associated ovarian carcinomas. The New England Journal of Medicine.

[ref-46] Yang S-Z, Wang A-Q, Du J, Wang J-T, Yu W-W, Liu Q, Wu Y-F, Chen S-G (2016). Low expression of ARID1A correlates with poor prognosis in intrahepatic cholangiocarcinoma. World Journal of Gastroenterology.

[ref-47] Zhang L, Wang C, Yu S, Jia C, Yan J, Lu Z, Chen J (2018). Loss of ARID1A expression correlates with tumor differentiation and tumor progression stage in pancreatic ductal adenocarcinoma. Technology in Cancer Research & Treatment.

