# Supplementary figures and images for: ARID1A alterations and their clinical significance in cholangiocarcinoma
Source: PeerJ. 2020 Dec 3;8:e10464. doi: 10.7717/peerj.10464 (PMC7719290; doi:10.7717/peerj.10464)

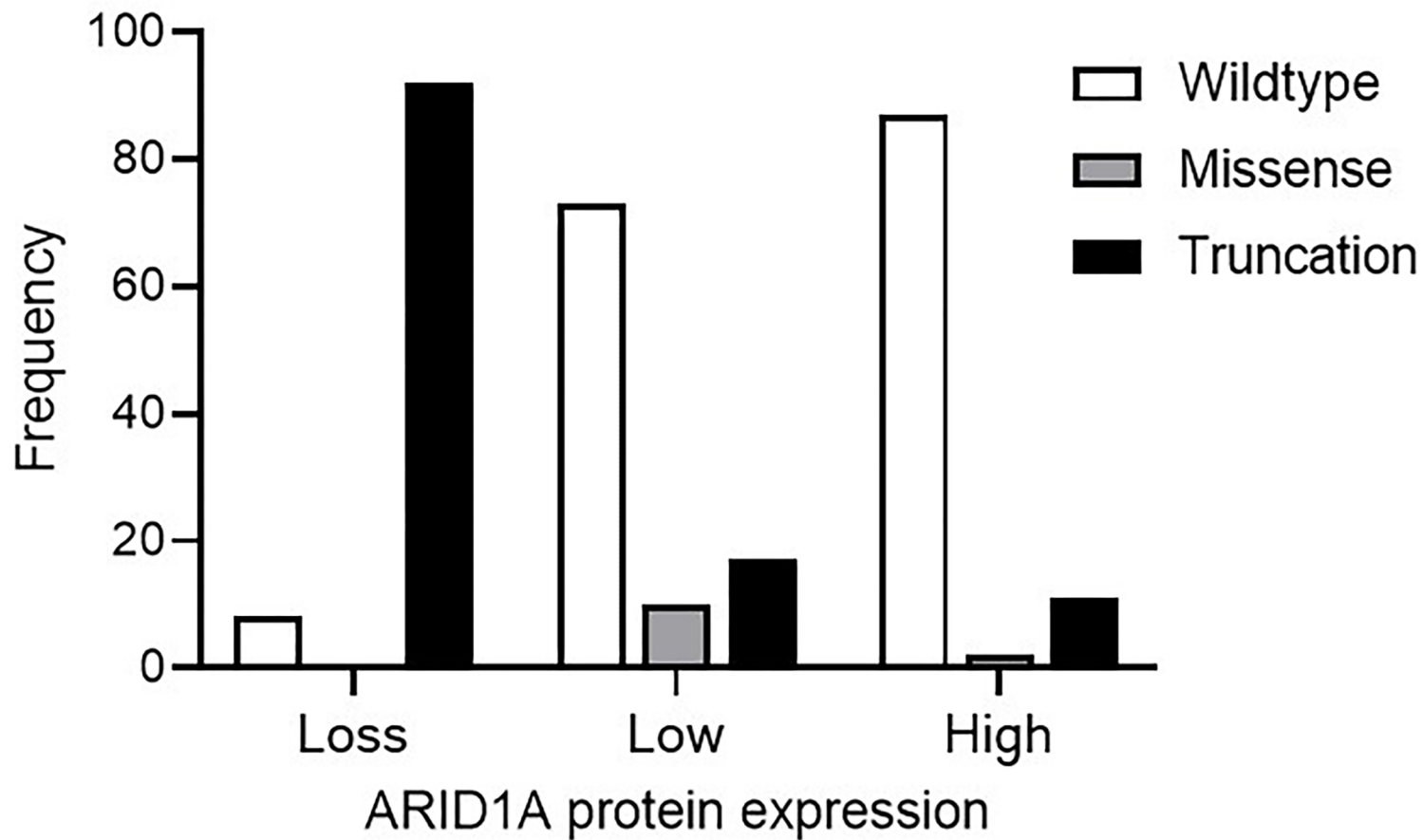

Supplement: Supplemental Information 5 — Wildtype denotes tumors with ARID1A wildtype, Missense denotes tumors with ARID1A missense mutation, Truncation denotes tumors with truncating ARID1A mutation. [file peerj-08-10464-s005.pdf]
